# Recent Advances in the Management of Clear Cell Renal Cell Carcinoma: Novel Biomarkers and Targeted Therapies

**DOI:** 10.3390/cancers15123207

**Published:** 2023-06-16

**Authors:** Valentina Schiavoni, Roberto Campagna, Valentina Pozzi, Monia Cecati, Giulio Milanese, Davide Sartini, Eleonora Salvolini, Andrea Benedetto Galosi, Monica Emanuelli

**Affiliations:** 1 Department of Clinical Sciences, Polytechnic University of Marche, 60020 Ancona, Italy; v.schiavoni@pm.univpm.it (V.S.); r.campagna@univpm.it (R.C.); v.pozzi@univpm.it (V.P.); moniacecati@gmail.com (M.C.); g.milanese@univpm.it (G.M.); a.b.galosi@univpm.it (A.B.G.); m.emanuelli@univpm.it (M.E.); 2 New York-Marche Structural Biology Center (NY-MaSBiC), Polytechnic University of Marche, 60131 Ancona, Italy

**Keywords:** renal cell carcinoma, clear cell renal cell carcinoma, biomarker, prognosis, therapy

## Abstract

**Simple Summary:**

The aim of the present review is to discuss novel prognostic and therapeutic markers for clear cell renal cell carcinoma, a subtype of renal cell carcinoma which is the most common variant and is characterized by high aggressiveness, invasiveness and metastatic potential, features that are responsible for the high mortality rate observed for this neoplasm. We firstly provide a background regarding the epidemiology and risk factors, and then, we focus on the established prognostic markers as well as novel ones. Subsequently, we analyze the recent advances in clear cell renal cell carcinoma treatment, and we discuss potential novel biomarkers for targeted therapy.

**Abstract:**

Renal cell carcinoma (RCC) belongs to a heterogenous cancer group arising from renal tubular epithelial cells. Among RCC subtypes, clear cell renal cell carcinoma (ccRCC) is the most common variant, characterized by high aggressiveness, invasiveness and metastatic potential, features that lead to poor prognosis and high mortality rate. In addition, diagnosis of kidney cancer is incidental in the majority of cases, and this results in a late diagnosis, when the stage of the disease is advanced and the tumor has already metastasized. Furthermore, ccRCC treatment is complicated by its strong resistance to chemo- and radiotherapy. Therefore, there is active ongoing research focused on identifying novel biomarkers which could be useful for assessing a better prognosis, as well as new molecules which could be used for targeted therapy. In this light, several novel targeted therapies have been shown to be effective in prolonging the overall survival of ccRCC patients. Thus, the aim of this review is to analyze the actual state-of-the-art on ccRCC diagnosis, prognosis and therapeutic options, while also reporting the recent advances in novel biomarker discoveries, which could be exploited for a better prognosis or for targeted therapy.

## 1. Introduction

Renal cell carcinoma (RCC) is classified among the 10 most common cancers in the world, accounting for 2–5 percent of all malignancies, particularly in the developed world, doubling the incidence in the United States of America [1,2]. RCC represents the most common cancer of the kidney and approximately 300,000 individuals worldwide are affected by this neoplasm. It is also responsible for over 10,000 deaths annually, thus representing the most lethal urological malignancy, displaying a 5-year survival rate of about 75% [3,4]. The risk in developing RCC increases with ageing, and the onset peak is around 70 years old [5]. The diagnosis of the disease at an initial stage is crucial for a favorable prognosis. Unfortunately, about 50% of all RCC cases are discovered incidentally, due to the lack of symptoms in the early stages of the disease. Moreover, 20% of patients are diagnosed as metastatic and more than 30% with localized cancer will develop distant metastasis following the complete resection of the primary tumor [6,7].

RCC is part of a heterogenous cancer group that originates from renal tubular epithelial cells [1]. Different subtypes were described, each one with different histology, genetic features and distinct response to therapy resulting in a variable clinical outcome. Most frequent RCC subtypes are clear cell (ccRCC, ~70–80% cases), papillary cell (pRCC, ~10–15% cases, and chromophobe renal cell carcinoma (chRCC, ~5% cases) [8]. Another rarer type of RCC is represented by that with sarcomatoid features [9,10].

In particular, ccRCC derives from epithelial cells of the proximal convoluted tubule in the nephron and it is histologically characterized by cells with clear cytoplasm [8]. ccRCC is the most common subtype and it accounts for a large part of mortality observed in RCC. ccRCC is hereditary for 2–3% of cases, particularly affecting subjects with an alteration of the von Hippel–Lindau (VHL) gene [11]. Indeed, ccRCC is characterized by a high proliferation rate compared to the other subtypes and it often metastasizes in the lungs, liver, bones and, for about 15%, in lymph nodes [12].

pRCC is classified as the second most frequent histologic subtype of RCC. Although most cases of pRCC are sporadic, it is subdivided in 2 hereditary forms: type 1 and type 2, depending on the stage, the grade and the prognosis of the disease. The type 1 is associated with a hereditary component which comprises MET mutations; type 2 is linked to mutations in fumarate hydratase gene, and it is associated with a worse prognosis [13,14].

chRCC is the third most common histologic subtype of renal cancer [15]. It is classified into two types: classical type and eosinophilic variant [16]. chRCC is characterized by positive prognosis, in fact it is considered less aggressive compared to other RCC subtypes, due to the low tendency to develop metastases [8].

Both pRCC and chRCC are less aggressive than ccRCC and display a better prognosis [12]. In this review, we will focus on ccRCC since it is the most aggressive subtype among the RCCs.

ccRCC is characterized by DNA alterations, including the copy number alterations (CNAs), as the loss of chromosome 3p and VHL inactivation, methylation and mutations, which are involved in tumor development and progression [17].

These molecular changes are responsible for pathway disruption and aberrant growth factor production, such as vascular endothelial growth factor (VEGF), platelet-derived growth factor (PDGF) and HIF pathways, which promote oncogenesis [18]. Invasiveness, ability to develop metastasis and chemoresistance are a major challenge in the treatment of most of neoplasms [19,20,21,22,23].

Thus, considering that ccRCC is characterized by high incidence and late diagnosis, tendency to metastasize and a remarkable chemoresistance, the research of new biomarkers for early diagnosis is of primary importance for a more accurate prognosis and for developing new targeted therapies for the management of this malignancy [24]. In fact, due to the combination of all these negative features, the survival rate for ccRCC is very low [24].

Even if several prognostic factors and diagnostic targets have been identified, the implementation of research is rapidly ongoing attempting to improve the ccRCC diagnosis, prognosis and therapy. In this review we will comprehensively describe the recent advances in ccRCC biomarker discoveries for diagnosis, prognosis and therapy.

## 2. Epidemiology

**Figure 1:**
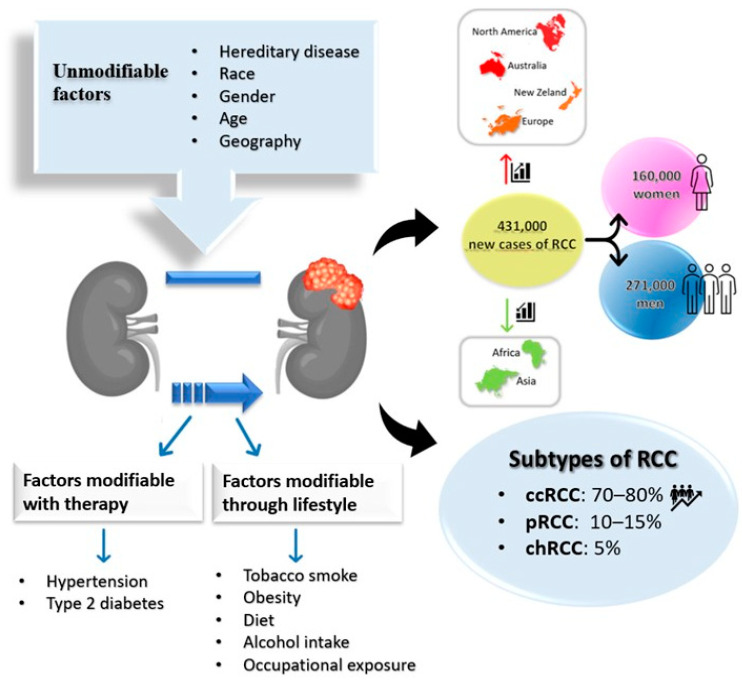
Risk factors, epidemiology and main subtypes of RCC. ccRCC: clear cell renal cell carcinoma; pRCC: papillary renal cell carcinoma; chRCC: chromophobe renal cell carcinoma.

RCC accounts for about 2% of cancer diagnoses and deaths worldwide; however, this number will increase over the years [25]. Kidney cancer has an ever-increasing incidence: 431,000 people a year have a diagnosis of RCC; 271,000 cases are diagnosed in men, while 160,000 are diagnosed in women, according to 2020 GLOBOCAN data [26], with a ratio of incidence between males and females of about 2:1 [27]. The highest incidence is observed in developed countries, while Asia and Africa have, on the contrary, a very low incidence, demonstrating that both race and lifestyle have a role in the incidence (Figure 1) [28]. Early diagnosis is very important, since the survival rate can change based on the disease stage [29].

Particularly, clear cell renal cell carcinoma is the most common histological subtype, representing 75–80% of all cases of RCC, and it is responsible for most of the morbidity and mortality of RCC [30]. It has the worst prognosis among all the most frequent RCC variants [31,32]. About 20–30% of patients have metastasis, and 30% of those with a localized carcinoma at an advanced stage will develop metastases [33]. The metastatic form is more aggressive, and it is related with a large mortality rate [34,35]. ccRCC can affect patients of different ages, but people between 60 and 70 years old are more likely to experience the disease than younger people [36]. It is diagnosed more often in males than in females—indeed, it is classified as the seventh most common malignancy among men. The survival rate is estimated at 60–70% after treatment in the initial stage of the disease, but the 5-year survival rate for advanced forms is poor with less than 10%, despite the progress in therapies [30,32]. Moreover, ccRCC has the highest mortality rate considering genitourinary cancers, and clinical outcomes are also not satisfactory due to drug resistance [37]. Thus, the identification of new molecular targets and the development of new therapeutic strategies are therefore of vital importance [34].

## 3. Risk Factors and Prevention

North America and Europe have the highest RCC incidence worldwide. The increasing incidence is likely due to improved detection by means of imaging techniques, but it could also depend on a higher prevalence of risk factors related to lifestyle such as obesity, hypertension and smoking [38]. Other factors that are considered related to the risk of developing RCC are age, sex, race and hereditary diseases [25,26]. Sporadic RCC is diagnosed in patients aged 65 to 74. Nevertheless, the average age is lower (44 years) among subjects affected by a hereditary disease, VHL disease, who have a 70% lifetime risk and represent 5% of all ccRCC cases [39]. Furthermore, pRCC diagnoses have a higher probability among subjects > 60 than ccRCC, while chRCC does not appear to be significantly age-related [2,40].

RCC is more common in males than in females, as well as for most other cancers. Moreover, the survival rate is lower in men [41]. This is probably associated with modifiable risk factors, such as smoking, hypertension and obesity. On the contrary, chRCC has a higher probability in women than ccRCC or pRCC [12,41].

Race is another risk factor: the incidence of RCC is variable in different ethnic groups in the United States, as shown in a study by Batai et al. demonstrating a higher incidence among African Americans, Hispanic Americans and Native Americans compared to White Americans [42]. In addition, the higher incidence of ccRCC among Caucasian Americans is also noticeable, while pRCC results are more common among African Americans. Moreover, these discrepancies are due not only to race differences, but also to the lack of health care, inability to access screening, limited access to cancer treatments and a poor quality of life [43].

As previously mentioned, hereditary diseases are also considered among risk factors. Phosphatase and tensin homolog (PTEN) hamartoma tumor syndrome/Cowden syndrome—associated with germline mutations in the PTEN gene—predispose individuals to various subtypes of RCC, including ccRCC, pRCC and chRCC. VHL disease is characterized by mutations in VHL gene on chromosome 3, mutations that are found in 90% of ccRCC and lead to a decrease of gene products and an increased expression of HIF-1,2 [44]. ccRCC is also linked with mutations in BRCA1-associated protein 1 (BAP1). Patients with somatic BAP-1 mutations have a high malignant form of ccRCC and the mean age of diagnosis is 40–45 years [45]. Other genetic syndromes linked with ccRCC are hereditary paraganglioma–pheochromocytoma syndrome (PGL/PCC), which involves mutations in the succinate dehydrogenase (SDH) gene, hyperparathyroidism-jaw tumor syndrome (HPT-JT), characterized by mutations in cell division cycle (CDC73) gene and tuberous sclerosis complex (TSC) [46,47,48].

Hereditary papillary renal cell carcinoma (HPRCC) is a familiar renal tumor syndrome characterized by a predisposition to the bilateral and multifocal development of type 1 papillary renal cell carcinoma. HPRCC is transmitted as a dominant character, and it is linked with mutations of the MET oncogene that can activate cell proliferation signaling and inhibition of apoptosis and promote cancer progression [49]. Hereditary lipomatosis and renal cell cancer (HLRCC) is a hereditary syndrome linked with type 2 pRCC, characterized by mutations in fumarate hydratase (FH) gene. The inactivation of FH gene leads to an increase of HIF-1, which promotes tumor metastasization [50]. BHD, caused by a germline mutation of the FLCN gene, is instead associated in particular with chRCC subtype, and it is less malignant than VHL-disease-associated ccRCC and HLRCC [16].

The risk factors listed until now are all unmodifiable, but even modifiable factors contribute to the development of RCC, such as tobacco smoking, alcohol consumption, eating habits and body weight. The primary prevention concerns the change of lifestyle with the aim of avoiding or reducing the onset or the development of the disease. Tobacco smoking is related with the risk of developing RCC, as well as other types of cancer [43]. In fact, tobacco smoke contains carcinogens—as beta-naphthylamine and nicotine—that are metabolized when they are filtered through the nephron, promoting inflammation and inducing DNA damage and thus, starting the carcinogenesis [2]. The risk of the development of RCC is greater in smokers, and it is related to the number of cigarettes smoked and the duration of smoking. In fact, a previous study demonstrated that regular smokers are 3.6 times more likely to develop the disease than non-smokers [51]. Instead, another study showed that the relative risk (RR) is lower in patients who have stopped smoking for 10 years [52]. The research article by Patel et al. is interesting, which revealed that smoking is an important risk factor for ccRCC and pRCC, but not for chRCC subtype [53].

Obesity is also related to the likelihood of developing kidney malignancies, and an association has been found between a higher body mass index (BMI) and RCC risk. According to a study from the European Prospective Investigation into Cancer and Nutrition (EPIC), a higher BMI is associated with a RR equal to 2.25 of RCC. The vitamin D and omega-3 trial (VITAL) study showed that a 5 Kg weight gain increases the risk by 25% in males and 35% in females [26,54]. Another study demonstrated that obesity represents a risk factor for ccRCC (RR = 1.8) and chRCC (RR = 2.2), while it is not associated with the development of pRCC [55]. Even diet could contribute to the development of RCC; in particular, the consumption of fruit and vegetables could have a protective effect and reduce RCC prevalence rate, while a diet rich in dairy and proteins is associated with a higher risk of RCC. However, the relationship between food and kidney cancer is not as strong as in other types of malignancies, such as gastric or colorectal cancer [56]. As concerns alcohol, a moderate intake is associated with a decreased risk of RCC, while heavy drinking leads to an increase of risk in both men and women [26].

Although RCC is not linked to a specific occupational exposure to substances, unlike bladder cancer and malignant mesothelioma, trichloroethylene (TCE) has been associated with kidney cancer, particularly with the ccRCC molecular subtype ccB, as well as with liver cancer and lymphoma [57]. TCE is a degreasing agent, and it is considered highly carcinogenic. It is found in the blood of 10% of US population, according to a study from the National Health and Nutrition Examination Survey (NHANES), and it seems to be able to cause DNA adducts, renal cell genotoxicity and cytotoxicity after its activation by the pathway involved in glutathione transfer in the liver and kidney [57]. Other chemicals such as benzene, vinyl chloride, herbicides and cadmium are indicated as agents that contribute to RCC development [58].

Hypertension and type 2 diabetes are considered associated with an increased risk of developing RCC. According to a study carried out in the US, the overall risk (OR) for RCC is doubled in patients with hypertension, but it was also demonstrated that the overall response rate (ORR) was higher in African Americans (ORR = 2.8) than in White Americans (ORR = 1.9) [59]. Moreover, a meta-analysis of 18 prospective studies found that patients with a history of hypertension have an increase of RCC risk of 67%, and each increase of 10 mm Hg in blood pressure was associated with 10–22% increased risk of kidney cancer [60]. The possible link between kidney malignancies and hypertension is not defined yet, but it could involve chronic renal hypoxia and lipid peroxidation due to the formation of reactive oxygen species (ROS). Indeed, oxidative stress is a key process involved in several pathological conditions, including cancer onset and progression [61,62,63,64,65,66]. Furthermore, the efficacy shown by antihypertensive agents against RCC metastasis, as shown in some basic and meta-analytic studies [67], is interesting. As concerns type 2 diabetes, its relationship with RCC is unclear. In the Nurses’ Health study, a significant association between these two types of disease was found—above all, a higher risk of RCC in women [68,69]. In addition, metabolic factors such as obesity, hypertension, diabetes and dyslipidemia have been shown to be related to the onset and development of ccRCC [70].

It would be of great importance to be able to make a diagnosis of ccRCC at an early stage, in order to improve the patient survival rate and to make treatment more effective; however, there are currently no screening programs for the early detection of ccRCC. A screening program could also be useful as an economic strategy to reduce the costs resulting from systemic therapies. Tumor markers with high sensitivity and specificity have not yet been validated for ccRCC screening, but some candidate molecules have been recently proposed, such as aquaporin 1 (AQP1) and perilipin 2 (PLIN2). Furthermore, DNA methylation, microRNAs (miRNAs) and long non-coding RNAs (lncRNAs) have been reported to be associated with some subtypes of RCC [71].

## 4. Diagnosis

Diagnosis of kidney cancer is incidental in 60% of cases. Localized RCC is generally asymptomatic, and indeed, the clinical examination allows only late diagnosis because the “classical triad” of symptoms, including flank pain, gross hematuria and palpable abdominal masses, are not very frequent [72]. Therefore, the discovery of disease occurs when the patient undergoes medical tests, such as ultrasound of the abdomen, for other reasons—25–30% of ccRCC cases are metastatic at initial diagnosis. RCC can give metastases in the lung (50–60% of cases), in the liver (30–40% of cases) and in bone, but also in the other kidney as well as in the adrenal, brain, spleen, gut and skin [73,74].

Laboratory examination of serum creatinine, hemoglobin, leukocyte and platelet counts, lymphocyte to neutrophil ratio, lactate dehydrogenase, C-reactive protein (CRP) and serum-corrected calcium should be ordered if RCC is suspected [72].

Diagnostic imaging techniques are essential for the diagnosis of kidney cancer. Usually, RCC and clinical benign cystic kidney are diagnosed by ultrasonography, and CT is used to evaluate local invasiveness, involvement of lymph nodes or distant metastases. Furthermore, MRI and thoracic, abdominal and pelvic CT with contrast medium are mandatory for accurate tumor staging in suspected malignant cases. Abdominal MRI and high-resolution CT scan without contrast medium are recommended in cases of allergy to CT contrast medium [12,75].

In the case of suspected malignant lesion, a renal tumor core biopsy is performed in order to have histopathological confirmation of malignancy. In particular, a biopsy is recommended before treatment with ablative therapies and in patients with metastatic disease before starting systemic treatment because it has high accuracy and furthermore, complications such as bleeding or tumor seeding are rare [76]. Moreover, through histopathological analysis it is possible to define the RCC subtype, since ccRCC shows different features compared to other variants; in particular, it is characterized by nested clusters of cells with a clear cytoplasm, surrounded by a dense endothelial network [8].

## 5. Prognostic Factors

Prognostic factors inform about patient outcomes regardless of medical and/or surgical treatment [77]. They are classified into anatomical, histological, clinical and molecular factors. The first two are supported by a higher level of evidence than clinical and molecular factors. Currently, the most used anatomical prognostic factor is the tumor, nodes and metastasis (TMN) staging system, proposed by the International Union Against Cancer and the American Joint Committee on Cancer and used as a prognostic system since 1977 for multiple solid tumors [6,78,79]. The TNM staging system defines local extension of the primary tumor, extension into the adrenal gland, extension beyond the renal capsule or Gerota’s fascia (T), involvement of regional lymph nodes (N) and spread to distant sites, showing the presence of distant metastases (M) [79]. In particular, the letter T, referring to the size of the primary tumor, is flanked by a scale ranging from 1 to 4, gradually defining tumors of larger dimensions. N indicates whether the cancer has extended to the lymph nodes and ranges from 0 to 3. M indicates metastases and can range from 0 to 1. Staging of renal cell carcinoma is mainly based on pathological examination and diagnostic imaging: stages I and II tumors, T1N0M0 ≤ 7 cm and T2N0M0 > 7 cm, respectively, are limited to the kidney, while stage III and IV include tumors that extend beyond the kidney [6].

In addition to TNM staging, several factors influence the prognosis of patients with ccRCC, among which the microscopical and macroscopical histopathological factors. Particularly, tumor grade, subtype, presence of sarcomatoid or rhabdoid features, tumor necrosis and microvascular invasion (MVI) are considered histological prognostic factors. The European Association of Urology (EAU) guidelines recommend the use of tumor grade and subtype [80]. Fuhrman and World Health Organization/International Society of Urological Pathology (WHO/ISUP) grading systems have been introduced to histologically classify RCC cells by means of morphological parameters. Through these systems, the lesion is classified according to the pathological stage, tumor dimensions, cell arrangement and type and nuclear grade. This latter turned out to be the most effective in predicting the development of metastases after nephrectomy [81]. The Fuhrman grading system is based on the assessment of nuclear size, shape and prominence in a four-level classification scheme. In 2012, ISUP reformed the Fuhrman system by basing the classification of tumors graded 1 to 3 on nucleolar prominence, allowing for less inter-observer variation, while grade 4 tumors are defined on the basis of nuclear pleomorphism, presence of giant cells, or sarcomatoid /rhabdoid differentiation. Furthermore, the percentage of sarcomatoid component is considered to be prognostic, a larger percentage being associated with a worse survival. This classification is recommended for ccRCC and pRCC, but not for chRCC [82]. The morphotype of RCC is another prognostic factor: clear cell subtype has an unfavorable outcome compared to pRCC and chRCC, but this difference is lost when tumor stage and grade are considered [78,82]. In fact, the prognosis is worse in all RCCs of higher stage and grade.

Tumor necrosis and microscopic vascular invasion are other prognostic factors. Once added into the ISUP classification system, the first one provided a greater predictive ability for cancer-specific survival with respect with the latter alone [83]. Tumor necrosis has been reported in 21–32% of ccRCC cases and is related to larger tumor size, higher grade and higher proliferative activity. This prognostic factor is associated with aggressive tumor behavior, thus affecting patient survival: 10-year cancer specific survival in grade 3 tumors without necrosis is in fact 62%, while it drops to 30% in grade 3 tumors with necrosis [83].

Metastatic spread of RCC can occur via both blood and lymph vessels. Microscopic vascular invasion (MVI) is linked to the presence of tumor cells within microscopic veins or lymphatic vessels and, in several retrospective studies, it is associated with inferior survival. MVI is more frequent in ccRCC than in non-ccRCC, recurring in 29% versus 12% of cases, respectively [6]. While macroscopic tumor invasion into the renal vein and the inferior vena cava has been recognized as a prognostic factor within TNM system for a long time, MVI has been only recently recommended as a prognostic tool by the ISUP. In a study of Bedke et al., MVI and lymphovascular invasion (LVI) were validated as prognostic factors for RCC, showing that they both correlate with low survival. Moreover, they have also been associated with advanced TNM stage, high Fuhrman grade and sarcomatoid features. In univariate analysis, they both correlate with cancer-free survival, while in a multivariate analysis. MVI was shown to be an independent prognostic factor. Microvascular and lymphovascular invasion are both related to metastatic spread and lower survival in ccRCC patients and are validated as prognostic factors for poor outcome [84]. Among macroscopical histopathological prognostic factors there are tumor invasion into perirenal tissues, macrovascular invasion into the renal vein and inferior vena cava, as mentioned above, and local lymph nodes. Tumors that exhibit these characteristics show an inferior progression-free and overall survival compared to stage I and II tumors [85].

Clinical factors can be considered prognostic and, among these performance statuses (PSs), presenting symptoms and paraneoplastic syndromes have been investigated. In addition, laboratory tests of serum creatinine, hemoglobin, leukocyte and platelet counts, lymphocyte to neutrophil ratio, lactate dehydrogenase, C-reactive protein and calcium are required [78]. These factors have been included in different prognostic scoring systems for risk assessment as they are prognostic for survival [72]. Independent prognostic factors have been combined to develop prognostic models to be used to predict cancer outcomes. Clinical and histopathological factors were added to the TNM system for the development of these prognostic models [78].

Particularly, for localized RCC, pre- and post-operative scores have been developed to assess patients’ prognosis. The first nomogram was developed in 2001 by Kattan et al. Later, the UCLA integrated staging system (UISS), the stage, size, grade and necrosis score (SSIGN), the Cindolo, the Leibovich, the Sorbellini and the Karakiewicz models were introduced. These models include all RCC subtypes, but only Leibovich introduced different algorithms for each histological variant; it was developed for use in ccRCC. Prognostic models and nomograms are mentioned in the EAU guidelines, but their use in routine clinical practice is not recommended [86,87]. The two most used are UISS and SSIGN. The latter is included in the guidelines and is used for patient stratification, mainly for therapeutic clinical trials and for evaluating the role of biomarkers in predicting patient survival. For risk assessment during cytokine treatment in metastatic disease, the Memorial Sloan Kettering Cancer Center (MSKCC) model proposed by Motzer et al. was the gold standard. This model evaluated as prognostic factors a poorer Karnofsky PS, absence of prior nephrectomy, high lactate dehydrogenase, low hemoglobin and high albumin-corrected calcium. Further refinement was obtained by Heng et al., who introduced the International Metastatic RCC Database Consortium (IMDC) score. In this system, the prognostic factors considered are six: Karnofky PS, time from diagnosis to treatment, hemoglobin, corrected calcium, platelet and neutrophil count. As in the MSKCC model, 3 risk groups were generated to stratify patients according to the overall survival: favorable, intermediate and poor, on the basis of the risk factors involved (0 factors; 1 to 2 factors; 3 to 6 factors, respectively) [78,88,89]. Currently, prognostic factors of IMDC are recommended for the management of metastatic ccRCC. IMDC may also be prognostic even after second-line treatment, although it has been developed in treatment-naïve patients.

Nowadays, no molecular prognostic factors are included in the above-mentioned prognostic models. Although genetic or other biomarkers are not routinely used to predict the prognosis of patients with RCC, in ccRCC several genetic alterations have been described that could improve current prognostic models. The identification of molecular markers is also useful to know better the RCC biology and to develop new targeted therapies [26,78]. Several molecular prognostic markers have been studied over the years. Many molecular changes associated with tumor development and progression have been described in ccRCC. The use of genomic, transcriptomic, and proteomic signatures could be a valid biomolecular approach for the evaluation of patient prognosis, but it is also useful for having an early diagnosis [4].

DNA mutations are frequent in each type of tumor, including ccRCC. Chromosomal abnormalities, particularly in chromosome 3 for ccRCC, have been associated with a worse prognosis. Furthermore, the most frequent mutational events occur in the short arm of chromosome 3, which includes VHL, PBRM1, BAP1 and SETD2 genes, and the loss of this arm is a frequent event in ccRCC. The resulting loss of heterozygosity leads to the loss of a copy of the above-mentioned genes. In ccRCC, mutations in PBRM1, BAP1 and SETD2 have been reported in 40%, 14% and 3% of cases, respectively. The first two have been related to an unfavorable prognosis [17]. Additionally, the gain of 7q and the loss of 9p, 9q and 14q are associated with a worse survival, suggesting alterations of genes involved in the progression of the tumor [90,91].

Among DNA alterations, the inactivation of VHL tumor suppressor gene has been observed in most patients with sporadic ccRCC. VHL gene deficiency results in the activation of HIF-1α and -2α, which in turn, leads to the upregulation of proangiogenic genes, including VEGF [92]. HIF-2α expression is increased in 75% of subjects affected by ccRCC, while the percentage drops to 38% in non-clear cell RCC patients. In particular, in ccRCC patients HIF-2α is associated with a more aggressive phenotype. However, VHL inactivation cannot be considered a prognostic factor since it is found in 80% of ccRCC cases. Nevertheless, it has been hypothesized that the loss of VHL function caused by gene mutations affects the progression of RCC [6,93]. Another effect caused by the inactivation of the VHL gene is the increase in VEGF concentration, which induces tumor angiogenesis. In ccRCC particularly, VEGF is related to the size of tumor, Fuhrman grade, tumor necrosis, tumor stage, MVI and RCC-specific survival. Moreover, downstream molecules of the VEGF pathway, such as phospho-extracellular signal-regulated kinase (pERK), could be used as biomarkers for response to therapy, although further confirmatory studies are needed [80].

Among the molecular prognostic factors studied are also Ki-67, p53 and PTEN [78]. Ki-67 is a cellular proliferation marker whose high expression is linked to higher recurrence rates and worse survival. Furthermore, it is associated with an aggressive phenotype of ccRCC [94]. p53 has controversial prognostic significance but it has been shown to be an independent predictor of metastasis-free survival in patients with localized ccRCC. Lastly, the loss of the tumor suppressor PTEN occurs during carcinogenesis, which is related to a worse prognosis in RCC [95,96].

Genetic signatures can help distinguish different risk groups in RCC. In particular, an expression panel of 34 genes, ClearCode34, was developed from the ccA/ccB classification in order to predict recurrence risk in patients with localized ccRCC, and it outperformed UISS and SSIGN systems in terms of discrimination. Both molecular subtypes of ccRCC were associated with recurrence-free survival (RFS), cancer-specific survival (CSS) and overall survival (OS) and ccA was found to correlate with a better survival than ccB [97,98]. Additionally, many different markers have been studied after the introduction of immunotherapy, to evaluate the response to immune checkpoint inhibitors used alone or in combination with cytotoxic T-lymphocyte antigen 4 (CTLA-4) inhibitors, tyrosine kinase inhibitors (TKI) or mTOR inhibitors. Programmed death-ligand 1 (PD-L1) is expressed on the surface of tumor cells, and it is involved in the mechanism of tumor immune evasion. PD-L1 positive tumor cells are associated with an inferior survival in ccRCC; the 5-year cancer-specific survival rate of these patients was in fact 42–47%, while PD-L1 negative patients have a greater percentage (66–83%). In addition, a higher expression of PD-L1 corresponds to a higher tumor grade and stage [99].

DNA methylation, miRNA and lncRNAs are epigenetic phenomena which can regulate gene expression in several cancerous and non-cancerous diseases [66,100,101,102]. RNA and cell-free DNA are potential non-invasive biomarkers for ccRCC because they can be detected in serum and urine samples. In ccRCC, an alteration in DNA methylation was observed in the pre-cancerous state and it was associated with high-grade tumors. Through cell-free methylated DNA immunoprecipitation and high-throughput sequencing, Nuzzo et al. were able to detect early tumors by non-invasive methods, using plasma and urine samples. A total of 300 differentially methylated regions have been identified and used to build a classifier in order to distinguish RCC samples from both control plasma and urine [103]. Moreover, a study by Minardi et al. shows a higher overall methylation rate in tumor than in healthy tissue. This group also studied histone acetylation, demonstrating an inverse pattern compared to methylation. A higher percentage of global methylation and a decrease in acetylation with increasing Fuhrman grade have also been observed, thus suggesting a correlation of these markers with tumor aggressiveness [104].

MicroRNA and long non-coding RNA are RNA molecules that are transcribed, but do not code for any protein. By binding to the complementary sequence in the 3′UTR region of mRNAs, they negatively regulate gene expression. The expression profile of miRNA is considered a tumor marker, since the alteration of their expression occurs due to chromosomal abnormalities, epigenetic mechanisms and mutations in the sequence of their DNA that affect their genomic position. Given that they can be detected in serum, plasma and urine samples by means of non-invasive procedures, miRNAs have gained relevance for ccRCC early diagnosis and prognosis [105].

One of the most studied and most important circulating miRNAs is miR-210. An elevated level of miR-210 was found in the serum of early-stage ccRCC patients, compared with healthy controls, thus proving its suitability as a biomarker for early detection. Moreover, it has been reported that miR-210 upregulation occurs in the presence of a hypoxic environment with accumulation of HIF-1α [106].

Along with miR-210, two other miRNAs were found upregulated in ccRCC: miR-1233 and miR-155. In addition, miR-210 and miR-1233 have been shown to be downregulated after surgery, thus proving to be good markers for cancer prognosis [105]. Lower levels of miR-206 and miR-122-5p were detected in the serum obtained from ccRCC patients, compared to controls, while an increase in these were observed in metastatic disease, thus identifying a correlation between enhanced miR-206 and miR-122-5p expression and shorter disease-free survival [107]. In addition to these lists, miR-193a-3p, miR-362 and mir-572 also showed an increase in ccRCC patient serum, while miR-28-5p and miR-378 were decreased compared to control groups. All these were detected in the initial phase of ccRCC, but some miRNAs have been described as associated with later phases, such as miR-451, which was found in plasma [108]. This one is downregulated in stage III and IV of ccRCC and it additionally correlates with a worse response to chemotherapy [108]. Urine samples represent another promising source for searching for miRNA biomarkers. Particularly, miR-210-3p is upregulated in urine samples of ccRCC patients and after surgery its levels were found to be decreased [109,110]. miR-122, miR-1271 and miR-15b were found to be increased in urine specimens [111]. miR-30c-5p, on the contrary, is lower in ccRCC patients than in healthy controls; in fact, it normally acts as a tumor suppressor in cancer by inhibiting the heat shock protein family A member 5 (HSAP5) gene, which is linked to cancer growth, aggressiveness and metastasis. Tumor suppressor miRNA promoter methylation could be an interesting new strategy to identify disease progression, as demonstrated with miR-30a-5p that is shown to be elevated in ccRCC and metastatic urine samples compared to non-metastatic ccRCC and healthy controls [111].

Long non-coding RNAs are also involved in the regulation of gene expression and are implicated in the development of various cancer types, including RCC. Several studies have identified an increasing number of lncRNAs that appear to have a regulatory role in cancer cell proliferation, migration, apoptosis and metabolism. lncRNAs can serve as serum biomarkers that can be used, like miRNAs, for early diagnosis or to predict the prognosis of patients, but also as therapeutic targets [112]. Wu et al. developed a panel comprising 5 ccRCC-dysregulates lncRNAs (lncRNA-LET, PVT1, PANDAR, PTENP1, linc00963) able to distinguish between ccRCC patients and healthy controls [113].

The patient’s prognosis appears to be related to aberrant expression of lncRNAs. lncRNA MALAT-1, for example, seems to be associated with tumor size and stage, lymph node metastases, and poor prognosis in ccRCC [114]. The overexpression of NEAT-1 demonstrated in ccRCC correlates with tumor size and grad, and lymph node metastases, showing a worsening in terms of overall survival. In addition, NEAT-1 knockdown has been shown to weaken cell proliferation, thus suggesting its suitability for the prediction of poor prognosis in ccRCC patients [115]. Among the upregulated lncRNAs, lnc-ZNF180-2, linc00152, HOTAIR and HEIRCC have been described. ATB and FTX are also upregulated and their inhibition lead to a reduction in cell migration and invasion capacity. H19, TCL6, CAM1-AS1, GAS5 and LOC389332 act as tumor-suppressors, and are instead, downregulated; in particular, H19 and TCL6 are negatively correlated with TNM stage, lymph node metastases and distant metastases [116].

lncRNAs also affect cancer cells apoptosis. It has been previously shown by Xiong et al. that a decreased lncRNA-ATB expression is able to promote apoptosis in renal cell carcinoma, thus demonstrating that its overexpression could hold back the apoptotic process during cancer development. CCAT1 is also involved in the regulation of apoptosis and its knockdown led to an increase in apoptotic RCC cells, as well as caspases 3, 7 and 9. An increase in oncogenic lnRNAs lowers the percentage of apoptotic cells, which is on the contrary, enhanced by the forced expression of tumor suppressor lncRNAs, such as GAS5 and TCL6 [117,118]. In addition, lncRNAs contribute to resistance to drugs used for the treatment of ccRCC. In particular, lncARSR was highly expressed in cells which show a resistance to sunitinib, while lncSRLR is upregulated in cells with intrinsic resistance to sorafenib. Xu et al. showed that the knockdown of this latter sensitized resistant cells, while its overexpression was able to confer drug resistance to reactive cells [119].

RAB17 belongs to RAB family and has been demonstrated to be associated with a variety of malignancies. In ccRCC, low expression levels of RAB17 have been found to be correlated with unfavorable clinicopathological features and with poor prognosis. Furthermore, RAB17 expression was demonstrated to be negatively correlated with immune cells infiltration, thus suggesting that RAB17 might be a potential prognostic biomarker for ccRCC patients and for estimating immunotherapy response [120].

Proteinuria, defined as an abnormal amount of protein in the urine, has been found in several studies to be an additional prognostic factor associated with the survival of kidney cancer patients after kidney surgery [121]. Indeed, the analyses of several studies show that proteinuria is a sensitive marker for the evaluation of the progression of chronic kidney disease in clinical practice. Several studies reported differences in OS at 5 years of follow up between patients with proteinuria compared to patients with absent proteinuria, with an increased OS observed for patients not affected by proteinuria (77% vs. 65%) [122].

Moreover, since proteinuria is a known marker of renal damage and an independent predictor of progression to the state of chronic kidney disease, the possibility should be considered that it should be evaluated to its level in the pre-operative setting in order to design a properly tailored treatment strategy for the specific patient [123].

## 6. Treatment

ccRCC is the most common subtype of RCC and represents 85 percent of all RCC tumors. Generally, surgery is the gold standard for localized renal cell carcinoma. In particular, the two most used methods are radical nephrectomy and nephron-sparing surgery. The latter, when technically feasible, is recommended for patients at stage T1 and T2 because it allows for the preservation of as much as possible of the normal ipsilateral renal unit. Instead, cytoreductive nephrectomy (CN) is indicated in patients with metastatic cancer, characterized by a giant primary tumor and few metastatic lesions. It has been observed that this technique can act synergistically with immunotherapy by removing cytokines and proteins that prevent the immune response [124]. CN is a controversial issue, as it has been shown that sunitinib—in combination with CN—was not superior to sunitinib alone, and therefore, requires further studies. For elderly patients with poor physical condition, tumor ablation is the most promising method because it has the advantage of being minimally invasive and requiring a short treatment time [54]. Instead, the metastatic ccRCC is treated with immunotherapy using interleukin 2 (IL-2) or interferon-α (IFN-α), which has remained the main treatment for more than 20 years, although response rates range from 15 to 25%. All subtypes of RCC are resistant to chemotherapy and radiotherapy, although some studies have shown that the latter could represent a promising therapeutic modality, especially stereotactic ablative body radiation (SAbR), which can be used for the treatment of early inoperable and metastatic RCC [125].

The lack of sensitivity to chemotherapy has stimulated the research of new treatment options (Table 1).

ccRCC is known as a highly vascular tumor, so antiangiogenic therapies represent the most sought-after approach. The introduction of therapeutic agents blocking angiogenesis by targeting VEGF pathway has been very important in the treatment of ccRCC. In particular, TKIs have been shown to be effective in ccRCC therapy [24,92]. As previously mentioned, ccRCC is characterized by inactivating mutations of the VHL gene. pVHL is the VHL gene product, which targets the α-subunit of the transcription factor HIF for degradation under normoxic conditions. HIF is composed of two subunits: HIF-α and HIF-β. In particular, HIF-2α promotes carcinogenesis and controls the expression of genes involved in angiogenesis, overregulating VEGF. When HIFα accumulates, due to the loss of VHL, it upregulates tumorigenic hypoxia-responsive genes, among which is VEGFA, whether there is enough oxygen or not. Since ccRCC express high levels of VEGFA, inhibitors of VEGF and its receptors are used to treat this type of disease. Among the drugs that block angiogenesis by acting against TK, sunitinib and pazopanib—which are used for first-line treatment in ccRCC after an improvement in progression-free survival (PFS)—have been demonstrated in pivotal studies comparing sunitinib with IFN-α and pazopanib with placebo [92]. In addition, a COMPARZ study demonstrated similar PFS between sunitinib and pazopanib. Recently, cabozantinib, another TKI, which acts against a broad range of targets such as VEGFR, MET and AXL, has been approved by the Food and Drug Administration (FDA) for first-line treatment in patients with advanced ccRCC. Cabozantinib was compared to sunitinib as initial therapy in a randomized phase 2 CABOSUN study, and it showed a prolonged PFS in patients with poor/intermediate-risk disease [126]. Lenvatinib is another TKI that blocks VEGFR, and it is used in combination with everolimus. This combination is better than either agent alone in previously treated patients [126]. Two additional TKIs are axitinib and sorafenib, but these are used as second-line agents after demonstrating an improvement in terms of PFS [24]. Bevacizumab is a monoclonal antibody targeting VEGFA approved in 2004. It has a consistent PFS benefit in combination with IFN-α in first-line treatments [127]. A novel inhibitor of VEGF receptor which has recently been approved by the US FDA is tivozanib, which is used particularly in patients who relapse after two or more prior systemic therapies [128].

Another therapeutic target is represented by HIF2α. Small-molecule inhibitors of HIF2α that allosterically disrupt its heterodimerization with HIF1β have been identified. In particular, PT2385 was the first inhibitor tested in humans and then improved by developing a second-generation small-molecule antagonist of HIF2α, belzutifan (MK-6482), which has received the FDA approval in August 2021 [129].

The mTOR signaling pathway represents an additional promising therapeutic target, since some ccRCCs show mutations in genes encoding components of mTOR pathway. mTOR is a serine/threonine kinase that forms two complexes: mTORC1 and mTORC2. The drugs everolimus and temsirolimus, which act by inhibiting mTORC1 and thereby decreasing the translation of mRNAs that encode proteins involved in cell survival and proliferation, and angiogenesis have been approved for the treatment of advanced ccRCC [130].

Further strategy to treat ccRCC is immunotherapy, which consists in the use of medicines in order to boost a person’s own immune system to recognize and destroy cancer cells more effectively. The immune system uses checkpoints, which are proteins on immune cells needing to be turned on (or off) to start an immune response. Kidney cancer cells use these checkpoints to avoid being attacked by the immune system. However, these drugs target the checkpoint proteins, thus helping to restore the immune response against cancer cells. For a long time, the cytokine IL-2 has been used in high doses, but due to its toxicity and the absence of predictive biomarkers, checkpoint inhibitors have been considered [92,131].

Checkpoint inhibitors target immune checkpoint receptors or ligands, among which PD-1 receptor and its ligands PDL-1/L2, and CTLA-4 receptor and its ligands CD80/86 that reduce T-cell activation and cause cancer cell immunotolerance. Pembrolizumab and nivolumab are drugs that target PD-1. Nivolumab was approved in 2015 after studies demonstrating an improvement in overall survival compared with everolimus in patients with metastatic ccRCC [132]. Pembrolizumab can be used in combination with axitinib or levantinib in first-line treatment [133]. Nivolumab could be instead employed as second-line treatment for patients with advanced ccRCC, but its combination with ipilimumab, an inhibitor of CTLA-4, can be effective for patients with intermediate or poor risk disease who have not received any treatment [126]. Additionally, the combination of nivolumab and cabozantinib might be used as the first treatment in people with advanced kidney cancer [134]. Avelumab is another PD-1 inhibitor that can be employed with axitinib as first treatment in patients with advanced ccRCC [135].

In the last 15 years, therapies targeting VEGF pathway have been the most used for the treatment of ccRCC; however, resistance to VEGF-targeted agents universally develops. For this reason, several studies have been performed in order to evaluate the combination of drugs, and the most promising ones are combinations of immune checkpoint inhibitors with VEGF inhibitors or with another immune checkpoint inhibitor, such as cabozantinib/nivolumab [92].

**Table 1:** Molecular targets and relative drugs used for ccRCC management.

Molecular Target	Drug	Reference
VEGFR	Sunitinib	[92]
	Pazopanib	[92]
	Cabozantinib	[126]
	Lenvatinib	[126]
	Axitinib	[24]
	Sorafenib	[24]
	Bevacizumab	[127]
	Tivozanib	[128]
HIF2α	PT2385	[129]
	Belzutifan (MK-6482)	[129]
mTOR	Everolimus	[130]
	Temsirolimus	[130]
Checkpoint inhibitors (PD-1 or CTLA-4)	Nivolumab (PD-1)	[132]
	Pembrolizumab (PD-1)	[133]
	Avelumab (PD-1)	[135]
	Ipilimumab (CTLA-4)	[126]

## 7. Novel Strategies

Recently, many efforts have been made to search for new diagnostic and prognostic molecules that can be used for early diagnosis and to predict the prognosis of patients with ccRCC. Another important goal of the search for new biomarkers is their potential use as new therapeutic targets.

Several conceivable biomarkers have been recently investigated, although they all require further validation studies before being adopted into clinical practice. However, these emerging biomarkers may be useful for understanding the mechanisms of drug resistance or for the development of new targeted therapies.

Many studies carried out to search for new molecular markers have been performed both on tumor tissue samples and on clear cell renal cell carcinoma cell lines.

The SPARC/osteonectin, CWCV and Kazal-like domains proteoglycan 1 (SPOCK1) is a proteoglycan which is involved in many types of cancer, including RCC. In particular, it plays a key role in cell proliferation, apoptosis, adhesion and migration, and its overexpression contributes to metastasis formation and drug resistance. Higher levels of both mRNA and protein have been demonstrated in ccRCC compared to healthy tissues, and this overexpression is related to more advanced clinical stage, larger tumor size, lymph node and distal metastases, as well as worse prognosis, with shorter OS and disease-free survival. In vitro and in vivo assays evidenced that knockdown and overexpression of SPOCK1 inhibited and potentiated the proliferative and metastatic capacity of ccRCC cells, respectively. SPOCK1 has also been shown to stimulate the epithelial–mesenchymal transition (EMT), thus promoting cancer cell invasion, correlating directly with the expression of mesenchymal markers and, inversely, with epithelial markers. In fact, its knockdown showed an increase in epithelial markers such as E-cadherin, thus suggesting a reduced progression of EMT, while an opposite effect was caused by SPOCK1 overexpression [136,137].

Extracellular matrix (ECM) modifications play a key role in several processes, including tissue repair and cell proliferation and motility [138,139,140,141]. Interestingly, SPOCK1 has been shown to be involved in the regulation of matrix-degrading metalloproteinases (MMPs), a family of proteolytic enzymes capable of degrading basement membrane and extracellular matrix components, being therefore involved in metastasization. In particular, MMP-2, MMP-9, MMP-14 and MMP-16 are required for EMT progression. In SPOCK1-depleted ccRCC cell line CAKI-1, MMP-2, -3 and -9 were found downregulated, while only MMP-2 was downregulated in SPOCK1-depleted 786-O ccRCC cell line, thus suggesting MMP-2 as a general target in ccRCC cells. Overexpression of SPOCK1 has been shown to contribute to an increase in MMP-2 enzymatic activity and in the expression of its upstream activators, such as MMP-14 and MMP-16, while the knockdown of SPOCK1 triggered their decrease. Therefore, secreted SPOCK1 induces the MMP-14/MMP-16-MMP-2 axis and because of this, promotes EMT, thus increasing the invasive capacity of ccRCC cells. The SPOCK1-MMP-2 axis is related to a worse prognosis and can be considered as a specific biomarker for the prediction of EMT-regulated invasion of ccRCC cells, as well as a possible therapeutic target [137].

Another study evaluated MMP-2, MMP-9 and CD44 as possible prognostic biomarkers. In particular, their immunohistochemical expression was analyzed in association with the histopathological subtype of RCC (302 total cases, of which 243 ccRCC and 59 non-ccRCC). CD44 binds to the extracellular matrix and acts as a platform for MMPs. Immunohistochemistry showed that there were no significant differences in CD44 expression between ccRCC and non-ccRCC cases, while MMP-2 and MMP-9 were more expressed in non-ccRCC cases. Moreover, MMP-2 overexpression was associated with a reduced risk of death in patients. However, only the ccRCC and CD44 subtypes were independent risk factors for patient death. A previous study showed that enhanced CD44 expression correlated with shorter OS in ccRCC, thus considering it as an independent risk factor capable of predicting recurrence-free survival, disease-specific survival and OS in patients with ccRCC. In addition, CD44 expression was positively associated with MMP-2 and MMP-9 in the ccRCC group, and correlated with nuclear grade, thus suggesting the contribution of these molecular markers to aggressiveness in ccRCC [142,143].

Another potential marker recently investigated is G2 and S-phase expressed 1 (GTSE1), which acts as an oncogene, promoting cell proliferation, migration and invasion, and regulating EMT. The GTSE1 protein is linked to the cell cycle and expressed in the G2 and S phases. It is found co-localized in microtubules and tubulin and plays a key role in chromosomal alignment. In ccRCC, mRNA and GTSE1 protein levels were higher than in normal tissues and cells, and related to poor patient survival. Through GTSE1 knockdown experiments, an inhibition of cell proliferation, migration and invasion and an activation of apoptosis have been demonstrated. GTSE1 works by regulating the expression of Krüppel-like factor 4 (KLF4), which is a tumor suppressor. KLF4 silencing has been shown to rescue the inhibition of cell migration caused by GTSE1 knockdown and to reverse EMT. GTSE1 expression levels are related to OS and disease-free survival, and furthermore, could be used as a novel biomarker for patient prognosis and as a new therapeutic target for ccRCC [144].

Molecule interacting with CasL-like protein 2 (MICALL2) is a cytoskeleton regulator that has been associated with the tumorigenic ability in many malignancies [145]. In particular, previous studies have shown that it promotes ccRCC malignancy by inducing EMT, such as SPOCK1 and GTSE1. MICALL2 is more expressed in ccRCC tissues and cell lines compared to healthy tissue and normal renal tubular epithelial cell lines and it was shown to have a predictive function in ccRCC carcinogenesis; in fact, MICALL2 overexpression led to enhanced cell proliferation, migration and invasion, while its knockdown had an opposite effect. Moreover, in vivo experiments have revealed that an increased expression of MICALL2 contributes to tumor growth and expansion. Similar to the others listed so far, it can therefore be considered a new prognostic marker and a potential therapeutic target [146].

Many other proteins, which are involved in the development and growth of ccRCC, have been studied in recent years; for example, A disintegrin and metalloproteinase-12 (ADAM12), which belongs to the type I transmembrane protein family. This protein has an oncogenic role, such as SPOCK1 and GTSE1, and its high expression is an indicator of poor overall survival. ADAMs are able to regulate cell adhesion, migration and signaling, as well as proteolysis. These proteins have different types of substrates, including cancer-related proteins, such as NOTCH receptor and ligand, epidermal growth factor receptor (EGFR) ligand, interleukin-6 receptor (IL-6R), tumor necrosis factor (TNF) and its receptor, E-cadherin and CD44 [147]. ADAM dysregulation is linked to the initiation and progression of tumors. In particular, ADAM12 is upregulated in both ccRCC tissues and cells and is correlated with gender, TNM stage and tumor grade. ADAM12 overexpression was shown to promote tumor growth and metastasis, while its knockdown had a reverse effect, thus inhibiting cell proliferation, migration and invasion. Being a secreted protein, it can be detected in blood and body fluids, and this makes it a good tumor marker candidate [148].

Spermatogenesis associated 18 (SPATA18) and EF-hand domain family member D1 (EFHD1), as opposed to SPOCK1, GTSE1 and ADAM12, are two tumor suppressors both involved in mitochondria functions. In particular, SPATA18, also called mitochondria-eating protein (MIEAP), is a p53-inducible protein which contributes to maintaining mitochondrial health by inducing lysosome-like organelles within mitochondria, thus leading to the elimination of oxidized mitochondrial proteins and improving mitochondrial functions. In a study by Lingui et al., SPATA18 was found to be overexpressed in ccRCC tissues compared to normal ones and its expression was associated with a better prognosis in ccRCC patients, thus suggesting its potential use not only as a diagnostic and positive prognostic biomarker, but also in ccRCC treatment, given its tumor suppressive function [149]. As concerns EFHD1, it is a mitochondrial enzyme that regulates calcium ions, which are second messengers and play a fundamental role in regulating cell functions. Mitochondria accumulate calcium ions to maintain their balance in the cytoplasm. Alterations in mitochondrial calcium influence malignant transformation and tumor progression. EFHD1 binds to the core of mitochondrial calcium uniporter (MCU), which is the main calcium transporter, through its N-terminal domain and this binding prevents the uptake of Ca^2+^ in the mitochondria. Since MCU levels are positively correlated with Ca^2+^ absorption, ROS production and propensity for metastatic dissemination, its inhibition, caused by EFHD1 binding, blocks Ca^2+^.absorption by mitochondria. In ccRCC tissues, EFHD1 was found downregulated at both mRNA and protein levels. Given that a reduced expression of EFHD1 was correlated with an unfavorable outcome, the enzyme is considered a promising prognostic factor [150].

Recent studies have shown that pathological behaviors of cancer cells, such as invasion and metastatic spreading, may also depend on the coordination of the membrane and actin cytoskeleton. ArfGAP with GTPase domain, ankyrin repeat and PH domain 2 (AGAP2) belongs to the AGAP family, including members involved in membrane and actin cytoskeleton changes, which are essential for normal physiological functions. Arf GTPase-activating proteins have been shown to be aberrantly expressed in several tumors and, in ccRCC, an overexpression of AGAP2 related to a reduction in overall survival has been demonstrated by means of immunohistochemistry. Moreover, overexpression is associated with clinical stage, poor prognosis and immune cell infiltration. AGAP2 may therefore be a valid prognostic biomarker. Given its ability to influence the abundance of immune cell infiltration, it can also become an important component for patients receiving precision cancer therapy [151].

Cytoskeleton-associated protein 2-like (CKAP2L) is a mitotic spindle protein encoded by the CKAP2L gene, whose mutations cause spindle organization defects. CKAP2L is associated with disease progression and prognosis in different types of cancer. Particularly, in ccRCC, its level of expression appears to be linked to the TNM stage and to a worse prognosis. In one study, it was suggested that its knockdown induces cell cycle arrest in the G2/M phase. In addition, since CKAP2L is correlated with numerous immune checkpoint inhibitor genes, it could indirectly affect immune system functions, thus favoring ccRCC development [152].

PICALM interacting mitotic regulator (PIMREG) is a mitotic regulator which has a key role in the metaphase-to-anaphase transition during the cell cycle. A high expression of PIMREG was evidenced in ccRCC by means of qRT-PCR. Moreover, its levels are positively correlated with tumor stage and grade, and thus, with a poor prognosis. In addition, knockdown experiments were performed, which demonstrated a significant inhibition of cell proliferation, migration and invasion and a slowing down of the cell cycle. PIMREG could therefore be used as a prognostic biomarker, and it could become a new potential therapeutic target [153].

Among the conceivable therapeutic targets in ccRCC, the enzyme nicotinamide N-methyltransferase (NNMT) has been shown to display a good potential as biomarker and therapeutic target. NNMT is a phase II drug metabolizing enzyme which methylates the nicotinamide to N1-methylnicotinamide [154]. Due to its role in nicotinamide homeostasis, its activity has a great impact on NAD^+^ homeostasis, NAD^+^-related enzymes and epigenetic regulation [63,155,156]. An overexpression of the enzyme has been observed in a number of solid malignancies, including gastric cancer, head and neck cancer, skin cancers, breast cancer, glioma and prostate cancer [157,158,159,160,161,162,163]. Remarkably, a NNMT overexpression has been reported in ccRCC compared to other renal cancers, and enzyme levels were significantly higher in small samples rather than large size tumors [164]. Furthermore, in another study, enzyme levels were associated with a poor prognosis of ccRCC patients [165]. Notably, due to enhanced differential expression between RCC subtypes and healthy tissue detected through several techniques such as immunohistochemistry, ELISA and proteomic analysis, NNMT has been proposed as a biomarker for the diagnosis of the main RCC subtypes, in combination with other markers [166,167,168]. Furthermore, several in vitro studies reported that NNMT downregulation was associated with a decreased proliferation and invasiveness in ccRCC cells [166,169,170,171,172]. In the light of these observations, the enzyme seems to be a promising diagnostic and prognostic biomarker, which could be also utilized as a therapeutic target due to the large number of NNMT inhibitors available and currently tested for cancer treatment [173,174,175,176,177,178].

Two other new molecules studied in the last period were found to be 2 potential immunotherapeutic targets: guanylate-binding protein 2 (GBP2) and B7-H3. GBP2 belongs to the family of interferon-induced GTPases and it was found to be highly expressed in several types of cancer with poor prognostic outcome. It has been shown in several studies that GBP2 models the immune microenvironment of the tumor and that its degree of expression is correlated with the rate of immune infiltration. In ccRCC, immunosuppressive cell infiltration represents a marker of altered therapeutic efficacy. Since not all patients respond to immune checkpoint blockade, the identification of reliable biomarkers of response to checkpoint block is vital to facilitate the improvement of the clinical efficacy of these therapies. In sarcomas, breast cancer and colorectal cancer, GBP2 shapes the immune microenvironment, and an elevated GBP2 expression is related to a favorable response to anti-PD1 therapy and to tumor-infiltrating T cells. Through the regulation of the tumor immune microenvironment, GBP2 can influence the prognosis of various malignancies. GBP2 expression is induced by interferon-γ (IFN-γ), which increases the expression of the PD-L1 ligand through the signal transducer and activator of transduction pathway 1 (STAT1), thus promoting tumor immune escape. By regulating PD-L1 expression through the STAT1 pathway, GBP2 promotes immune evasion, and it therefore indicates a worse prognosis for ccRCC patients. In addition to being a negative prognostic marker, GBP2 can be considered a potential immunotherapy target in ccRCC [179]. B7-H3 is instead a member of the B7 family of proteins and is an immune checkpoint molecule expressed in the immune microenvironment, in both cancer and immune cells. As concerns the tumor context, B7-H3 has been associated with tumor cell proliferation, metastasization and resistance to therapy. Normal and tumor tissues have shown a substantial difference in protein expression levels, thus suggesting the suitability of B7-H3 as a therapeutic target. In the cited study, no correlation was found between the expression levels of CTLA-4 and B7-H3, which was instead associated with PD-L1 expression. Moreover, immunohistochemistry showed a poor correlation between B7-H3 and PFS. B7-H3 could be important for the development of new immunotherapy treatments, as immunotherapy using the B7-H3 pathway is effective with the simultaneous use of both chemotherapy and radiotherapy [180].

The high frequency of occurrence of ccRCC in male patients compared to females could in part be explained by the mounting evidence that androgen-receptors function as an oncogene in ccRCC, fostering progression and hematogenous metastasis. This observation led to the hypothesis that the use of anti-androgen receptors could represent a possible therapeutic strategy to be explored [181,182,183].

## 8. Conclusions

ccRCC is characterized by high aggressiveness, invasiveness and metastatic potential, features that are associated with chemoresistance and radioresistance and which are responsible for the poor prognosis and high mortality rate of this malignancy. In this review, we highlighted the most novel biomarkers that could contribute to better prognosis assessment, as well as promising molecular markers which could be exploited for targeted therapies. Nonetheless, it is important to pursue the identification of new biomarkers with high precision and sensitivity in order to improve the outcome of ccRCC patients.
